# Comparative Phenotypic and Genotypic Analysis of *Erysipelothrix rhusiopathiae* Strains Isolated from Poultry

**DOI:** 10.3390/antibiotics15010011

**Published:** 2025-12-20

**Authors:** Ádám Kerek, Gergely Tornyos, Eszter Kaszab, Enikő Fehér, Ákos Jerzsele

**Affiliations:** 1 Department of Pharmacology and Toxicology, University of Veterinary Medicine Budapest, H-1078 Budapest, Hungary; tornyos.gergely@student.univet.hu (G.T.); jerzsele.akos@univet.hu (Á.J.); 2 National Laboratory of Infectious Animal Diseases, Antimicrobial Resistance, Veterinary Public Health and Food Chain Safety, University of Veterinary Medicine Budapest, H-1078 Budapest, Hungary; kaszab.eszter@univet.hu (E.K.); feher.eniko@univet.hu (E.F.); 3 One Health Institute, University of Debrecen, Nagyerdei krt. 98, H-4032 Debrecen, Hungary; 4 Department of Microbiology and Infectious Diseases, University of Veterinary Medicine, István u 2, H-1078 Budapest, Hungary; 5 National Laboratory of Virology, Szentágothai Research Centre, University of Pécs, H-7624 Pécs, Hungary

**Keywords:** *Erysipelothrix rhusiopathiae*, poultry, minimum inhibitory concentration, next-generation sequencing

## Abstract

**Background**: *Erysipelothrix rhusiopathiae* is an important zoonotic pathogen in poultry, yet little is known about its antimicrobial resistance (AMR) dynamics in avian hosts. With growing concerns about subtherapeutic antimicrobial use in animal agriculture, poultry-origin isolates represent a potential but under-characterized reservoir of resistance genes. **Methods**: We phenotypically tested 38 *E. rhusiopathiae* strains isolated from geese, ducks, and turkeys in Hungary (2024) using broth microdilution against 18 antimicrobial agents, following Clinical Laboratory Standards Institute (CLSI) guidelines. Nineteen phenotypically resistant strains were selected for whole-genome sequencing (Illumina platform), followed by de novo hybrid assembly, gene annotation (Prokka, CARD, VFDB), mobile element detection (Mobile Element Finder), and phylogenetic inference (autoMLST). **Results**: All isolates were susceptible to β-lactams, including penicillin, amoxicillin, and third-generation cephalosporins. Resistance to tetracyclines (up to 10.5%) and florfenicol (5.3%) was most frequently detected. Genomic analysis revealed the presence of *tetM* (9/19), *tetT* (2/19), and *erm(47)* (2/19) genes, all associated with chromosomally integrated mobile elements, ICE Tn6009 and IS ISErh6. Phylogenomic analysis demonstrated tight clustering into four clades, suggesting clonal expansion. Notably, one strain harbored a 64.8 kb genomic island carrying *ermC*, the first such finding in poultry-derived *E. rhusiopathiae*. **Conclusions**: Our data highlights the early emergence of mobile AMR determinants in *E. rhusiopathiae* from poultry and suggests that horizontal gene transfer may drive resistance even in chromosomally encoded contexts. The genomic stability and phylogenetic homogeneity of avian isolates underscore the need for targeted AMR surveillance in poultry sectors to mitigate potential zoonotic transmission risks.

## 1. Introduction

Antimicrobial resistance (AMR) is an increasingly critical concern for both animal and public health worldwide [1]. It is well established that the use of antimicrobial agents contributes to the spread of resistance, and the global demand for these agents is projected to rise substantially in the coming years, especially in the developing world, where livestock production is becoming increasingly intensive [2,3]. This trend has intensified the search for partial or complete alternatives to antibiotics, including antimicrobial peptides [4], essential oils and plant extracts [5,6], propolis [7,8,9], probiotics [10,11], and other promising bioactive compounds [12,13]. These approaches are complemented by improved vaccination protocols and rigorous implementation of biosecurity measures [14,15]. Responsible antibiotic use also hinges on evidence-based drug selection guided by pharmacokinetic and pharmacodynamic (PK/PD) data [16].

In the poultry sector, most AMR-related data concern *Escherichia coli* [17], *Enterococcus* spp., and *Staphylococcus aureus* [18]. *Erysipelothrix rhusiopathiae* is a rod-shaped [19], Gram-positive, facultatively anaerobic bacterium [20], primarily known as a swine pathogen [21], with 30–50% of healthy pigs acting as asymptomatic carriers via lymphoid tissues of the gastrointestinal tract [22]. Importantly, it also affects poultry—especially waterfowl—and is capable of zoonotic transmission through contact with feces, saliva, or other secretions of infected animals [19]. In humans, *E. rhusiopathiae* most commonly causes erysipeloid, a localized cutaneous infection characterized by painful, well-demarcated erythematous lesions, typically affecting the hands or fingers. Occupationally exposed individuals, including farmers, veterinarians, slaughterhouse workers, and fishermen, are at highest risk. Although rare, invasive human infections such as septicemia and endocarditis have been reported and are associated with substantial morbidity and mortality [23]. The pathogen is environmentally robust: it can survive in soil for up to 35 days, for 12 days under direct sunlight, and for months in buried carcasses [24].

In waterfowl production, *E. rhusiopathiae* causes significant disease burden, especially in geese, and to a lesser extent in ducks [25]. It has recently emerged as a concern in caged laying hens, particularly under outdoor housing conditions [26]. In laying flocks, outbreaks often present acutely, with mortality rates reaching up to 60% [27]. In waterfowl, mortality ranges from 1% to 50%, depending on how rapidly antimicrobial treatment is initiated [27]. Clinical signs include anorexia, depression, weakness, ruffled feathers, and drooping wings. Post-mortem findings typically include splenomegaly, hepatomegaly, and disseminated petechial hemorrhages consistent with sepsis [28]. Transmission has been linked to the poultry red mite (*Dermanyssus gallinae*) in attic-housed flocks [29]. In Sweden, repeated outbreaks from 2010 to 2019 prompted the licensing and field use of an inactivated vaccine in turkeys [30]. In Japan, a study showed that 30% of chicken meat samples from slaughterhouses were contaminated with *E. rhusiopathiae* [31], and severe outbreaks have also been reported in turkey populations [32].

During outbreak management, antimicrobial therapy remains the cornerstone of treatment, highlighting the need for continuous surveillance of *E. rhusiopathiae* resistance profiles. However, data on antibiotic resistance phenotypes and genotypes of poultry-derived isolates causing sepsis remain limited. Existing studies frequently report resistance to tetracyclines and fluoroquinolones, though multidrug-resistant (MDR) strains have not been commonly described [33]. Resistance to lincosamides, macrolides, and pleuromutilins has also been documented. Reported resistance genes include *tetM*, *lnuB*, *lsaE*, *ant(6)-Ia*, *aph-A3*, *spw*, *ermT*, *ermA*, *msrD*, *gyrA*, and *parC* [34,35,36].

The present study aims to characterize the phenotypic antimicrobial susceptibility profiles of *E. rhusiopathiae* strains isolated from clinically affected poultry. Additionally, using next-generation sequencing (NGS), we analyze the antimicrobial resistance gene content of these strains and identify associated mobile genetic elements.

## 2. Results

### 2.1. Phenotypic Resistance Profiles

The distribution of minimum inhibitory concentration (MIC) values for 13 antimicrobial agents across the *E. rhusiopathiae* isolates is presented in Table 1. The broadest MIC ranges were observed for tylosin, enrofloxacin, ceftriaxone, cefotaxime, and clindamycin. Most isolates exhibited low MIC values for penicillin, amoxicillin, cefotaxime, ceftriaxone, tylosin, clindamycin, and enrofloxacin, with the majority of results falling well below the respective clinical breakpoints. The exact MIC values are listed in Supplementary Table S1.

For oxytetracycline, 10.5% of isolates exhibited MIC values ≥ 16 µg/mL, exceeding the established susceptibility threshold. In the case of florfenicol, 94.7% of isolates showed MIC values ≤ 8 µg/mL, while the resistant subset (5.3%) displayed markedly higher MICs, far above the defined cutoff. Tylosin and tiamulin resistance was detected in a small proportion of isolates (7.9% and 7.9%, respectively). For clindamycin and enrofloxacin, the vast majority of isolates fell within the susceptible range, with only isolated cases showing elevated MICs.

The MIC_50_ and MIC_90_ values were generally low across most tested antimicrobials, particularly among β-lactam antibiotics (e.g., penicillin: MIC_90_ = 0.03 µg/mL; amoxicillin: MIC_90_ = 0.125 µg/mL). In contrast, higher MIC_90_ values were observed for doxycycline, oxytetracycline, florfenicol, and tiamulin (ranging from 4 to 16 µg/mL).

**Figure 1:**
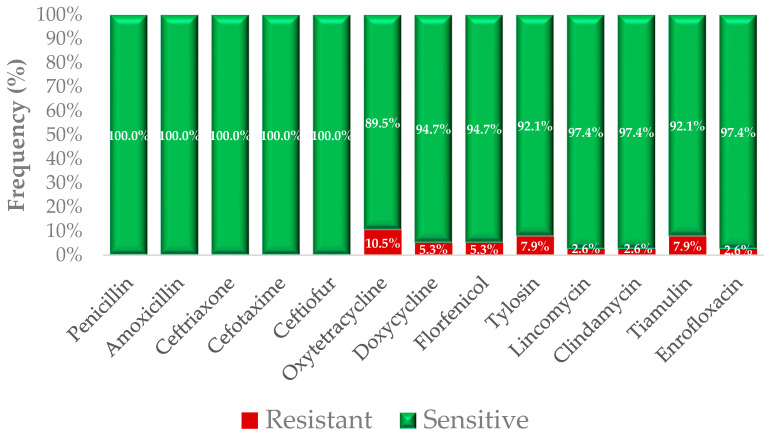
Antimicrobial susceptibility profile of *Erysipelothrix rhusiopathiae* isolates (*n* = 38) based on minimum inhibitory concentration (MIC) testing. The chart displays the proportion of susceptible (green) and resistant (red) isolates to 13 different antibiotics, as determined by broth microdilution. Susceptibility classification was based on established clinical breakpoints provided by the Clinical Laboratory Standards Institute (CLSI) or equivalent sources where available.

Antimicrobial susceptibility of the *E. rhusiopathiae* isolates was assessed using broth microdilution for 13 antibiotic agents for which clinical breakpoints were available (Figure 1). All tested isolates (100%) were classified as susceptible to penicillin, amoxicillin, ceftriaxone, cefotaxime, and ceftiofur based on their respective MIC threshold values.

For doxycycline, 84.7% of isolates were susceptible, while 5.3% exhibited resistance. Susceptibility rates for florfenicol and tylosin were both 94.7%, with 5.3% of isolates classified as resistant. In the case of lincosamide-class agents, 97.4% of isolates were susceptible to lincomycin and clindamycin. Susceptibility to tiamulin and enrofloxacin was 92.1% and 97.4%, respectively. The highest proportion of resistant isolates was observed for oxytetracycline (10.5%).

For imipenem, linezolid, ciprofloxacin, trimethoprim–sulfamethoxazole (1:19), and vancomycin, no official clinical breakpoints are currently available. Nevertheless, MIC values were determined for these agents to provide an overview of their in vitro activity against *E. rhusiopathiae*. The distribution of MIC values for each compound is presented in Table 2.

### 2.2. Correlation Between Phenotypic Resistance and Resistance Gene Carriage

To evaluate the functional relevance of the detected resistance genes, phenotypic antimicrobial susceptibility data were systematically compared with the genomic resistance profiles of the sequenced isolates. This integrated analysis enabled assessment of genotype–phenotype concordance across major antimicrobial classes.

To assess genotype–phenotype concordance, phenotypic antimicrobial susceptibility data were compared with the genomic resistance profiles of the 19 sequenced isolates. Tetracycline resistance determinants were detected in 11 isolates, including *tetM* in nine strains (strain IDs: 62478, 15312, 20293, 16538, 80414, 80120, 28661, 20241, 20242) and *tetT* in two strains (strain IDs: 57513, 99790).

All isolates exhibiting elevated MIC values to doxycycline or oxytetracycline (≥16 µg/mL) carried at least one tetracycline resistance gene, indicating complete concordance between phenotypic resistance and genomic detection for tetracyclines. Conversely, isolates lacking *tet* genes consistently displayed low MIC values within the susceptible range. Fisher’s exact test confirmed a significant association between the presence of *tet* genes and phenotypic tetracycline resistance (*p* < 0.05).

Macrolide resistance was observed in two isolates showing elevated tylosin MIC values, both of which harbored the *erm(47)* gene (strain IDs: 57513, 99790). No macrolide resistance phenotype was detected among *erm(47)*-negative isolates, supporting a strong genotype–phenotype correlation for macrolide resistance as well.

Overall, these findings demonstrate a high level of concordance between the genomic resistome and phenotypic resistance profiles, supporting the functional relevance of the detected resistance determinants.

### 2.3. Genomic Resistance Determinants

Among the 19 sequenced *E. rhusiopathiae* strains, the *tetM* gene was detected in 9 isolates, all showing 100% coverage and >99% identity. Additionally, two isolates carried the *tetT* gene with 100% coverage and 93.1% identity; both of these strains also harbored the *erm(47)* gene, with complete coverage and identity exceeding 99%.

All resistance genes were located on the chromosome, yet each was associated with mobile genetic elements (MGEs). The *tetM* genes were embedded in integrative and conjugative elements (ICEs) identified as the Tn6009 transposon, which has previously been linked to tetracycline, macrolide, and aminoglycoside resistance in other bacterial species. In contrast, the *tetT* and *erm(47)* genes were found within insertion sequence (IS) elements classified under the ISErh6 family, which appears to be specific to *E. rhusiopathiae*.

**Figure 2:**
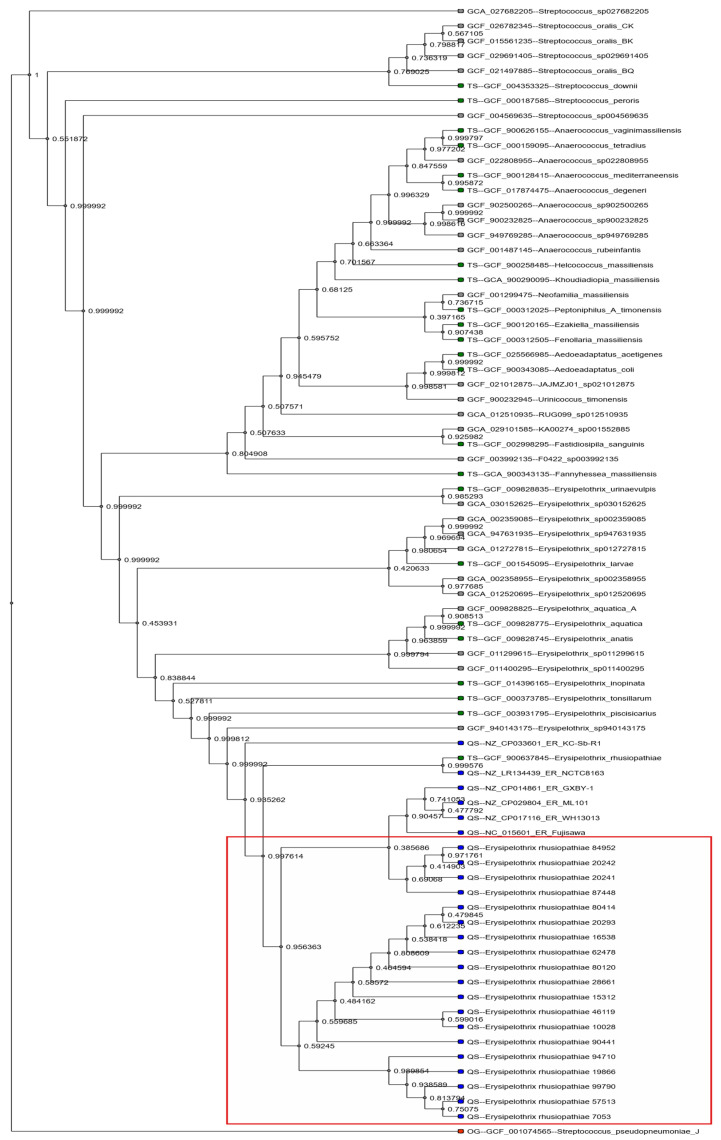
Phylogenetic tree of the sequenced *Erysipelothrix rhusiopathiae* isolates (*n* = 19) based on whole-genome comparisons. The isolates formed four well-separated clusters within a monophyletic lineage, distinct from other *Erysipelothrix* species and closely related genera (e.g., *Streptococcus*, *Anaerococcus*). Hungarian isolates from this study are highlighted in blue and enclosed in red boxes, while previously published strains are shown in gray. The tree was constructed using a whole-genome-based phylogenomic pipeline, and external taxa were incorporated to provide broader evolutionary context.

A phylogenetic tree was generated based on whole-genome alignments of the 19 sequenced isolates using the AutoMLST2 automated pipeline. The resulting tree revealed tight clustering among the isolates, with four well-defined clades forming a genetically homogeneous lineage (Figure 2). In addition to the study isolates, the tree included phylogenetically related Gram-positive taxa, such as *Erysipelothrix tonsillarum*, which were automatically incorporated by the software to enhance phylogenetic context and rooting.

Among the study isolates, Clusters 1 (80414, 20293, 16538, 62478, 80120, 28661, 15312, 90441) and 2 (94710, 19866, 99790, 57513, 7053) contained the majority of strains, while Clusters 3 (84952, 20242, 20241, 87448) and Cluster 4 (46119, 10028) formed smaller but distinct subgroups. The overall genomic similarity among the isolates suggests a shared evolutionary background, possibly linked to common geographic origin or host species.

IslandViewer analysis identified at least one genomic island (GI) in each of the 19 sequenced *E. rhusiopathiae* genomes. A conserved ~19.7 kb region was detected in 11 isolates, frequently associated with mobile genetic elements such as ICEs or ISs. This region contained *groS* and *groL* chaperonin genes along with multiple hypothetical proteins. Structural variation was observed, ranging from ~11 kb to 23 kb, but the core components were largely conserved. None of these islands carried known antimicrobial resistance or virulence genes.

In the remaining 8 isolates, unique GI architectures were observed. Several of these regions also contained IS elements (e.g., ISErh6), while others included genes linked to stress response or metabolism, such as *yjaB* and *dps2*.

A particularly noteworthy finding was a 64,833 bp mobile island identified in strain 99790. This complex region harbored the *ermC* macrolide resistance gene and represented the only genomic island among the sequenced isolates to contain an antimicrobial resistance determinant.

## 3. Discussion

The antimicrobial susceptibility profiles of the examined *E. rhusiopathiae* isolates were generally favorable, particularly in the case of β-lactam antibiotics, where all strains proved to be susceptible to penicillin, amoxicillin, and third-generation cephalosporins (cefotaxime, ceftriaxone, and ceftiofur; Table 1, Figure 1). These findings are consistent with previous studies reporting universal susceptibility to penicillin and amoxicillin in *E. rhusiopathiae* isolates [37]. A separate investigation similarly confirmed full susceptibility to penicillin, amoxicillin, and amoxicillin-clavulanate among all tested strains [33].

Among tetracycline-class antibiotics, resistance was observed in 10.5% of isolates against oxytetracycline and in 5.3% against doxycycline (Figure 1). Although tetracycline resistance has been described in this species before—for example, a 1984 Japanese study reported a 43% resistance rate to oxytetracycline [38]—more recent studies have also confirmed a strong association between tetracycline resistance and the presence of *tetM* genes, particularly in isolates of swine origin [39]. Given the limited data available for poultry-derived isolates, our findings underscore the need for heightened attention to emerging resistance in this sector.

For florfenicol, 94.7% of isolates were susceptible, while 5.3% displayed resistance. Considering the widespread veterinary use of florfenicol, even a low prevalence of resistance may carry clinical significance. A similar resistance frequency (7.9%) was observed for both tiamulin and tylosin—the latter being commonly used for respiratory infections in poultry (Figure 1). Regarding lincosamides (lincomycin, clindamycin) and fluoroquinolones (enrofloxacin), the vast majority of isolates were susceptible, with only a few strains exhibiting elevated MIC values (Table 2). These findings are in agreement with a Polish study in swine-derived isolates, which reported universal susceptibility to β-lactams, macrolides, and florfenicol, while resistance was already emerging to tetracyclines and enrofloxacin [34].

Taken together, our phenotypic susceptibility data support the continued efficacy of penicillin-based treatment as the first-line choice for *E. rhusiopathiae* infections in poultry. However, the presence of resistant subpopulations—especially against doxycycline and florfenicol—highlights a potential decline in therapeutic efficacy and underscores the importance of periodic, targeted susceptibility testing. This is particularly critical in host species and husbandry systems with elevated zoonotic potential.

Importantly, the phenotypic susceptibility patterns showed a high level of concordance with the genomic resistance determinants identified in this study (Figure 1; Table 1). All isolates exhibiting elevated MIC values to tetracyclines carried at least one tetracycline resistance gene (*tetM* or *tetT*), while macrolide resistance was exclusively associated with the presence of *erm(47)*. This tight genotype–phenotype correlation supports the functional relevance of the detected resistance genes and suggests that resistance in these poultry-derived isolates is primarily driven by well-defined, conserved genetic mechanisms rather than by transient or inducible phenotypes. From a One Health perspective, such stable resistance architectures may facilitate persistence and dissemination across animal populations and potentially into the human interface.

Importantly, comparison of phenotypic susceptibility data with the genomic resistome revealed a high degree of concordance between observed resistance phenotypes and detected resistance determinants. Isolates exhibiting elevated MIC values to tetracyclines were consistently associated with the presence of *tetM* or *tetT* genes, supporting the functional relevance of these determinants in poultry-derived *E. rhusiopathiae*. Conversely, isolates lacking tetracycline resistance phenotypes did not harbor known tetracycline resistance genes, indicating limited background or cryptic resistance in this population.

The genomic identification of *tetM* and *tetT* genes (Supplementary Table S2)—particularly in combination with *erm(47)*—points toward an expanded potential for antimicrobial resistance, possibly driven by the selective pressures of subtherapeutic antibiotic usage in the poultry sector.

Although the identified resistance genes were exclusively chromosomally located, their association with mobile genetic elements (MGEs), specifically Tn6009 (an integrative and conjugative element, ICE) and ISErh6 (an insertion sequence, IS), suggests the potential for mobilization. This raises the possibility of horizontal gene transfer both within *E. rhusiopathiae* populations and potentially to closely related pathogenic taxa.

Similarly, the low prevalence of phenotypic resistance to macrolides and lincosamides corresponded well with the limited detection of macrolide resistance genes, with *erm(47)* and *ermC* identified only in a small subset of isolates. This genotype–phenotype agreement further supports the biological relevance of the detected resistance loci.

The presence of Tn6009 has previously been reported in *Streptococcus suis*, *Enterococcus faecium*, and *Staphylococcus* species, where it has been associated with the carriage of *tetM*, *ermB*, and *aphA3* resistance genes [40,41]. Although ISErh6 remains less well-characterized, existing studies suggest it can be endogenously activated in *E. rhusiopathiae*, with its transpositional activity increasing in response to antibiotic exposure [42].

The consistent association of these mobile genetic elements with phenotypically expressed resistance suggests that horizontal gene transfer–linked resistance in *E. rhusiopathiae* is not merely genomic background noise but contributes directly to clinically relevant resistance profiles.

While chromosomal localization of resistance genes generally reduces the likelihood of classical plasmid-mediated transfer, the presence of MGEs—especially Tn6009 and ISErh6—points to genome-stabilizing and adaptive mechanisms that may facilitate more rapid population-level dissemination of resistance under selective antibiotic pressure. These findings underscore the need for continuous AMR monitoring in *E. rhusiopathiae* strains isolated from poultry. From a One Health perspective, these findings are particularly relevant, as *E. rhusiopathiae* is a recognized zoonotic pathogen capable of causing occupational infections in humans, most notably erysipeloid, endocarditis, and systemic septicemia. Resistance determinants such as *tetM* and *erm*-family genes identified in poultry-derived strains have also been reported in human clinical *E. rhusiopathiae* isolates, suggesting the existence of a shared or overlapping resistome across animal and human reservoirs.

Whole-genome sequence–based phylogenetic analysis of the 19 *E. rhusiopathiae* isolates revealed tight clustering, forming a distinct monophyletic group in comparison to other closely related bacterial species included in the tree (Figure 2). This genetic homogeneity supports the species-level identity of the isolates and further suggests that they likely originated from populations with shared ecological or epidemiological backgrounds. The automatic inclusion of other *Erysipelothrix* species—such as *E. tonsillarum*—as well as partially characterized strains, provided a valuable taxonomic context, strengthening the reliability of phylogenetic placement and species identification. Notably, whole-genome–based phylogenetic analyses of human clinical *E. rhusiopathiae* isolates reported in the literature have revealed close genetic relatedness to animal-derived strains, including those originating from swine and poultry. The tight clustering and limited genomic divergence observed in our study are therefore consistent with a pathogen population structure capable of crossing host species barriers, reinforcing the zoonotic and public health relevance of these lineages.

Our isolates formed four well-delineated visual clusters, which may reflect host-specific or geographic segregation. However, the relatively minor genetic divergence observed among them also suggests a common evolutionary origin. This pattern aligns with the known background of the isolates, as most were derived from waterfowl and collected during the same diagnostic period. The inclusion of other bacterial taxa in the tree was not incidental but was algorithmically implemented by the comparative analysis pipeline to enable more accurate calibration of evolutionary distances.

The phylogenetic homogeneity observed among the studied *E. rhusiopathiae* strains not only supports accurate species identification but also suggests a conserved genetic background for the detected resistance determinants—particularly *tetM* and *erm(47),* which appear to be inherited via the same family of mobile genetic elements (Tn6009 and ISErh6). The observed correlation between phylogenetic structure and resistome composition implies that these are not isolated resistance events, but rather components of a potentially stable, horizontally transmissible genomic context. This is consistent with earlier studies of porcine *E. rhusiopathiae* isolates, where a link between the carriage of *tet* genes and phylogenetic clustering was demonstrated [39], and positions our findings as the first to highlight similar dynamics in poultry-derived strains.

IslandViewer-based analyses confirmed the frequent presence of conserved MGE-associated genomic regions across the *E. rhusiopathiae* genomes examined. These islands likely represent evolutionarily stabilized horizontal gene transfer (HGT) acquisitions. One recurrent feature—a ~19.7 kb region carrying the *groS*/*groL* chaperonin operon and multiple hypothetical proteins—was found in most isolates. Although this region did not encode known resistance or virulence factors, its association with ICE or IS elements in several strains suggests potential for mobility. Similar conserved islands have been described in other Gram-positive animal pathogens; for example, a 17 kb ICE in *Streptococcus suis* has been shown to carry the *tetM* gene [43], reinforcing the notion that MGEs play a common role in shaping host-adapted bacterial genomes.

In a subset of strains (e.g., 90441 and 94710), we identified unique genomic island (GI) structures, several of which harbored insertion sequences such as ISErh6, and genes related to stress response or metabolism (e.g., *yjaB*, *dps2*). These elements illustrate the capacity of *E. rhusiopathiae* for genomic plasticity and acquisition of novel regions. This dynamic genomic architecture is in line with models of adaptive genome evolution in bacteria, wherein clade-specific mobile elements may accelerate genomic shifts under antimicrobial pressure [33].

One particularly notable finding was the identification of a >64 kb mobile genomic island in strain 99790, which represented the only isolate in this study harboring a macrolide resistance gene (*ermC*) embedded within a large, structurally complex genomic island. Although tetracycline resistance genes (*tetM* and *tetT*) were detected in multiple isolates, these genes were associated with smaller integrative or insertion sequence–based mobile elements, whereas strain 99790 uniquely carried a clinically relevant macrolide resistance determinant within a large putatively transferable genomic island. The detection of mobile resistance elements in poultry-derived *E. rhusiopathiae* strains gains additional significance in a One Health context, as macrolide resistance genes located on mobile genomic islands have previously been described in human clinical isolates of Gram-positive pathogens. This raises concerns that food-producing animals may act as silent reservoirs of transferable resistance determinants with zoonotic potential, particularly in occupationally exposed populations.

These findings suggest that, although relatively rare, large AMR-associated genomic islands can emerge within *E. rhusiopathiae* populations and may contribute to the dissemination of resistance across ecological and host boundaries. To our knowledge, this study represents the first report of a macrolide resistance gene located on a large mobile genomic island in poultry-derived *E. rhusiopathiae*, underscoring the importance of targeted genomic surveillance to mitigate potential animal and public health risks.

This study represents the first detailed characterization of genomic islands in *E. rhusiopathiae* strains isolated from poultry, highlighting their diversity, MGE-associated architecture, and occasional resistance gene content, as well as their conservation across a subset of the population.

## 4. Materials and Methods

### 4.1. Sampling and Identification of Erysipelothrix rhusiopathiae Strains

The strains included in this study were collected in 2024 by staff at the National Food Chain Safety Office (Hungary) reference laboratory. All isolates originated from deceased poultry submitted for diagnostic necropsy, including geese (32/38; 84.3%), ducks (2/38; 5.2%), and turkeys (4/38; 10.5%). Post-mortem examinations were performed by licensed poultry health veterinarians, and the bacteriological isolations were carried out by trained laboratory technicians.

Preliminary species identification was based on colony morphology, Gram staining, motility testing, and catalase activity. These were further complemented by biochemical profiling using the API Coryne system (Biomérieux Hungária Kft., Budapest, Hungary). Species confirmation was subsequently achieved using matrix-assisted laser desorption ionization–time of flight (MALDI-TOF) mass spectrometry (Flextra-LAB Kft., Budapest, Hungary) and the Biotyper software version 12.0 (Bruker Daltonics GmbH, Bremen, Germany; 2024 release).

Our research group received the pure cultures of the isolates for downstream phenotypic and genotypic analyses under an inter-institutional collaboration agreement. The isolates were stored in Microbank cryovials (Pro-Lab Diagnostics, Richmond Hill, ON, Canada) at −80 °C. Metadata associated with each strain included host species and the organ of origin (e.g., liver, lung).

### 4.2. Determination of Minimum Inhibitory Concentration

The selection of antimicrobial agents for susceptibility testing was designed to encompass a broad range of antibiotic classes with relevance to veterinary clinical practice, antimicrobial resistance surveillance, and public health. In addition to compounds commonly used for the treatment of *E. rhusiopathiae* infections in poultry, antibiotics of importance in food-producing animals and human medicine were included in order to support a One Health–oriented assessment of phenotypic resistance patterns.

The phenotypic antimicrobial susceptibility profiles of the *E. rhusiopathiae* strains were assessed by determining the minimum inhibitory concentrations (MICs), following the guidelines of the Clinical and Laboratory Standards Institute (CLSI) [44]. As no species-specific clinical breakpoints are currently available for *E. rhusiopathiae*, antimicrobial susceptibility interpretation was based on a combination of CLSI guidelines and peer-reviewed literature, following accepted practice for infrequently isolated veterinary pathogens. Breakpoints for penicillin, amoxicillin (extrapolated from ampicillin), ceftiofur, cefotaxime (extrapolated to ceftriaxone), enrofloxacin, and clindamycin were adopted from CLSI VET06 [45].

For tylosin, lincomycin, and tiamulin, doxycycline, oxytetracycline, and florfenicol resistance thresholds were derived from previously published studies investigating *E. rhusiopathiae* [34].

Revival of the isolates was carried out from −80 °C Microbank cryopreservation vials (Pro-Lab Diagnostics, Richmond Hill, ON, Canada). For MIC testing, isolates were suspended in 3 mL cation-adjusted Mueller-Hinton broth (CAMHB; VWR International, Debrecen, Hungary) supplemented with 5% defibrinated horse blood (Biolab Zrt., Budapest, Hungary) and incubated at 37 °C for 18–24 h. Susceptibility testing was performed using 96-well microtiter plates (VWR International, Debrecen, Hungary).

Each well was filled with 90 µL of CAMHB supplemented with 5% defibrinated horse blood, resulting in a final test volume of 100 µL per well after inoculation. Antimicrobial stock solutions (Merck KGaA, Darmstadt, Germany) were prepared at 1024 µg/mL according to CLSI recommendations [46]. Penicillin and amoxicillin (pH 7.2, 0.01 mol/L), as well as imipenem (pH 6, 0.1 mol/L), were dissolved in phosphate buffer. Cefotaxime, ceftriaxone, ceftiofur, doxycycline, oxytetracycline, tylosin, tiamulin, lincomycin, linezolid, clindamycin, and vancomycin were dissolved in distilled water.

For the potentiated sulfonamide combination, trimethoprim and sulfamethoxazole were prepared separately and subsequently combined at a fixed ratio of 1:19 (trimethoprim/sulfamethoxazole, *w*/*w*), in accordance with CLSI recommendations for testing potentiated sulfonamides. Sulfamethoxazole was dissolved in hot distilled water with the addition of 100 µL of 2.5 mol/L NaOH, while trimethoprim was dissolved in distilled water containing 100 µL of 0.05 mol/L HCl. The combined solution was then used as a single antimicrobial agent for the preparation of the dilution series. Enrofloxacin and ciprofloxacin were dissolved in distilled water with 100 µL of 1 mol/L NaOH. Florfenicol was dissolved in distilled water with 100 µL of 95% ethanol.

For MIC determination, a twofold serial dilution was prepared directly in the microtiter plate by transferring 90 µL of antimicrobial stock solution into the first column, followed by sequential twofold dilutions across the plate. After completion of the dilution series, excess volume was discarded after the 10th column, leaving 90 µL of antimicrobial-containing medium in each well. Bacterial suspensions were adjusted to a turbidity equivalent to 0.5 McFarland standard using a nephelometer (ThermoFisher Scientific, Budapest, Hungary). Ten microliters of the standardized bacterial suspension were added to each well, yielding a final inoculated volume of 100 µL per well and an approximate final inoculum of 5 × 10^5^ CFU/mL.

MIC values were determined using the Sensititre SWIN automated MIC reader (ThermoFisher Scientific, Budapest, Hungary), with interpretation via the VIZION software (version 3.4, 2024). Quality control was ensured by including the *E. rhusiopathiae* reference strain (ATCC 19414) in every testing run.

### 4.3. Next-Generation Sequencing

Based on phenotypic antimicrobial susceptibility results, 19 *E. rhusiopathiae* isolates showing resistance were selected for whole genome sequencing (WGS). Genomic DNA was extracted using the Zymo Quick-DNA Fungal/Bacterial Miniprep Kit (Zymo Research, Irvine, CA, USA), following the manufacturer’s instructions. Mechanical cell disruption was performed using bead-beating with a Qiagen TissueLyzer LT (Qiagen GmbH, Hilden, Germany) at 40 Hz for 5 min. Purified DNA was stored at −20 °C until further use.

For library preparation, the Illumina Nextera XT DNA Library Preparation Kit (Illumina, San Diego, CA, USA) was used. DNA fragmentation and indexing were carried out using the Nextera XT Index Kit v2 Set A with i5 and i7 index primers. DNA samples were diluted to a final concentration of 0.2 ng/µL in a 2.5 µL volume, followed by the addition of 5 µL Tagment DNA buffer and 2.5 µL Amplicon Tagment Mix. The tagmentation reaction was incubated at 55 °C for 6 min using an Eppendorf Mastercycler nexus GX2 thermal cycler (Eppendorf SE, Hamburg, Germany), and then cooled to 10 °C.

To stop the reaction, 2.5 µL of Neutralize Tagment buffer was added, and the mixture was incubated at room temperature for 5 min. The tagmented DNA was subsequently amplified using 7.5 µL Nextera PCR Master Mix and 2.5 µL each of i5 and i7 index primers. The PCR protocol included an initial denaturation at 95 °C for 30 s, followed by 12 cycles (95 °C for 10 s, 55 °C for 30 s, 72 °C for 30 s) and a final elongation step at 72 °C for 5 min, ending with a hold at 10 °C.

Indexed libraries were purified using the Geneaid Gel/PCR DNA Fragments Extraction Kit (Geneaid Biotech, New Taipei City, Taiwan) via column-based separation. DNA quantification was performed fluorometrically using the Qubit dsDNA HS Assay Kit (Thermo Fisher Scientific, Waltham, MA, USA). The individually indexed libraries were pooled in equimolar ratios for downstream sequencing.

Paired-end sequencing was performed by Novogene (Beijing, China) using the Illumina NovaSeq X Plus platform [47,48].

### 4.4. Bioinformatic Analysis

Raw sequencing data quality was initially assessed using FastQC v0.11.9 [49], Fastp v0.23.2-3 [50] and Bloocoo v1.0.7 [51]. These tools enabled the detection and partial correction of adapter contamination, base composition bias, and low-quality sequence regions. Cleaned and quality-filtered reads were subjected to de novo assembly using two independent assemblers: MEGAHIT v1.2.9 [52] and SPAdes v4.0.0 [53]. The resulting assemblies were merged using GAM-NGS v1.1b [54] to obtain a more robust and comprehensive draft genome sequence.

Assembly quality and completeness were evaluated using QUAST v5.2 [55] and BUSCO v5 [56]. Core genome metrics—such as genome size, sequence coverage, and heterozygosity—were estimated with GenomeScope v2.2 [57] based on *k*-mer distribution analysis. These metrics guided downstream parameter settings and confirmed the technical reliability of sequencing.

Open reading frames (ORFs) were predicted using Prokka v1.14.5 [58]. Antimicrobial resistance genes (ARGs) were identified using both Resistance Gene Identifier (RGI) v5.1.0 and ABRicate [59] leveraging the Comprehensive Antibiotic Resistance Database (CARD) [60]. Only resistance genes showing ≥80% sequence identity and ≥80% coverage against the CARD were retained for further analysis. Virulence-associated genes were screened using the Virulence Factors Database (VFDB) [61].

To determine the mobility of ARGs, MobileElementFinder v1.0.3 [62] was employed. A gene was classified as mobile if located within the maximal species-specific distance to a known mobile genetic element (MGE), as defined in the integrated database. PlasFlow v1.1 [63] was used to assess the potential plasmid origin of contigs. For both MGEs and plasmids, only hits located within a maximum distance of 10,000 base pairs were considered. Taxonomic identity was verified using CheckM v1.2.2 [64] and Kraken v1.1.1 [65]. Genomic islands (GIs) were predicted with IslandViewer 4, using the *E. rhusiopathiae* WH13013 complete genome as reference and default settings [66].

Whole-genome-based phylogenetic trees were generated via the autoMLST platform under default parameters. The pipeline automatically selected the most appropriate set of housekeeping genes, performed multiple sequence alignments, and constructed a phylogeny using the maximum likelihood method. Branch support was calculated via bootstrapping, and values are shown adjacent to tree nodes [67].

### 4.5. Statistical Analysis

Statistical analyses were performed using non-parametric methods due to the limited sample size. Differences in MIC distributions between antimicrobial classes were assessed using the Kruskal–Wallis test. Associations between phenotypic resistance and the presence of resistance genes were evaluated using Fisher’s exact test or Mann–Whitney U test, as appropriate. Statistical significance was defined as *p* < 0.05. Analyses were performed using R (version 4.1.0).

## 5. Conclusions

This study provides an integrated phenotypic and genomic characterization of poultry-derived *E. rhusiopathiae* strains, revealing overall preserved susceptibility to β-lactam antibiotics, alongside emerging resistance to tetracyclines and florfenicol in a subset of isolates. The detection of *tetM*, *tetT*, and *erm(47)* genes associated with mobile genetic elements highlights the presence of potentially transferable resistance determinants within avian populations. Whole-genome phylogenetic analysis demonstrated a genetically homogeneous population structure, suggesting a shared evolutionary background of the circulating strains. Notably, the identification of a large genomic island harboring *ermC* represents the first such report in poultry-derived *E. rhusiopathiae*, indicating ongoing genomic adaptation under antimicrobial pressure. Collectively, these findings underscore the importance of continued AMR surveillance in avian reservoirs and support the integration of genomic monitoring into One Health frameworks to mitigate potential zoonotic risks.

## Figures and Tables

**Table 1:** Frequency distribution of minimum inhibitory concentrations (MICs) for *Erysipelothrix rhusiopathiae* isolates (*n* = 38) obtained from poultry. Vertical red lines indicate clinical breakpoints. Gray background denotes empty (non-applicable or missing) data fields.

Antibiotics	Breakpoint	0.001	0.002	0.004	0.008	0.016	0.031	0.063	0.125	0.25	0.5	1	2	4	8	16	32	64	128	256	512	1024	MIC_50_	MIC_90_
	(µg/mL)																							
Penicillin	0.25	25	3	1	0	1	4	4															0.001	0.031
		65.8%	7.9%	2.6%	0.0%	2.6%	10.5%	10.5%																
Amoxicillin	0.5				2	5	15	9	7														0.031	0.125
					5.3%	13.2%	39.5%	23.7%	18.4%															
Ceftiofur	8										1	35	1	1									1	1
											2.6%	92.1%	2.6%	2.6%										
Ceftriaxone	2	12	1	7	5	5	1	3	0	1	1	2											0.004	0.063
		31.6%	2.6%	18.4%	13.2%	13.2%	2.6%	7.9%	0.0%	2.6%	2.6%	5.3%												
Cefotaxime	2	22	1	1	6	2	0	0	0	2	1	3											0.001	0.25
		57.9%	2.6%	2.6%	15.8%	5.3%	0.0%	0.0%	0.0%	5.3%	2.6%	7.9%												
Oxytetracycline	16										1	8	10	8	7	1	1	1	1				2	8
											2.6%	21.1%	26.3%	21.1%	18.4%	2.6%	2.6%	2.6%	2.6%					
Doxycycline	16							1	2	1	2	12	11	5	2	1	1						2	4
								2.6%	5.3%	2.6%	5.3%	31.6%	28.9%	13.2%	5.3%	2.6%	2.6%							
Florfenicol	32											2	17	3	11	3	0	0	1	1			2	16
												5.3%	44.7%	7.9%	28.9%	7.9%	0.0%	0.0%	2.6%	2.6%				
Lincomycin	16										1	30	5	1	0	0	1						1	2
											2.6%	78.9%	13.2%	2.6%	0.0%	0.0%	2.6%							
Tylosin	1	1	4	1	18	1	2	1	2	5	0	2	1										0.008	0.25
		2.6%	10.5%	2.6%	47.4%	2.6%	5.3%	2.6%	5.3%	13.2%	0.0%	5.3%	2.6%											
Tiamulin	32										1	22	10	2	0	0	1	2					1	4
											2.6%	57.9%	26.3%	5.3%	0.0%	0.0%	2.6%	5.3%						
Clindamycin	1	1	5	0	1	14	3	4	3	3	3	1											0.015	0.25
		2.6%	13.2%	0.0%	2.6%	36.8%	7.9%	10.5%	7.9%	7.9%	7.9%	2.6%												
Enrofloxacin	1	16	8	0	3	0	2	1	5	2	0	0	1										0.002	0.125
		42.1%	21.1%	0.0%	7.9%	0.0%	5.3%	2.6%	13.2%	5.3%	0.0%	0.0%	2.6%											

**Table 2:** Frequency distribution of minimum inhibitory concentration (MIC) values for *Erysipelothrix rhusiopathiae* isolates (*n* = 38) derived from poultry, tested against antimicrobial agents without established clinical breakpoints. Gray background indicates empty cells.

Antibiotics	0.001	0.002	0.004	0.008	0.016	0.031	0.063	0.125	0.25	0.5	1	2	4	8	16	32	64	128	256	512	1024	MIC_50_	MIC_90_
	(µg/mL)																						
Imipenem		1	0	2	3	0	1	0	1	23	7											0.5	1
		2.6%	0.0%	5.3%	7.9%	0.0%	2.6%	0.0%	2.6%	60.5%	18.4%												
Linezolid						10	0	0	0	9	18	1										0.5	1
						26.3%	0.0%	0.0%	0.0%	23.7%	47.4%	2.6%											
Ciprofloxacin	24	0	0	8	0	1	1	1	1	2												0.001	0.063
	63.2%	0.0%	0.0%	21.1%	0.0%	2.6%	2.6%	2.6%	2.6%	5.3%													
Potentiated sulphonamide ^1^	1	1	6	6	2	7	3	2	3	5	2											0.031	0.5
	2.6%	2.6%	15.8%	15.8%	5.3%	18.4%	7.9%	5.3%	7.9%	13.2%	5.3%												
Vancomycin																	27	0	8	0	3	64	256
																	71.1%	0.0%	21.1%	0.0%	7.9%		

^1^ trimethoprim sulfamethoxazole in 1:19 ratio.

## Data Availability

The datasets used and/or analyzed during the current study are available from the corresponding author on reasonable request. The sequencing files are available at https://www.ncbi.nlm.nih.gov/bioproject/PRJNA1356264, accessed on 2 November 2025.

## Supplementary Materials

The following supporting information can be downloaded at: https://www.mdpi.com/article/10.3390/antibiotics15010011/s1, including additional and supplementary data (*E. rhusiopathiae*). Supplementary Table S1: The exact MIC values of *Erysipelothrix rhusiopathiae*; Supplementary Table S2: Detected antimicrobial resistance genes.
